# Large In-Plane Tensile Deformation of a Novel Pre-Wound Six-Ligament Chiral Structure

**DOI:** 10.3390/ma18245514

**Published:** 2025-12-08

**Authors:** Naixin He, Yanping Song, Pengfei Huang, Jiachen Zeng

**Affiliations:** China Academy of Space Technology (Xi’an), Xi’an 710199, China; he_naixin@126.com (N.H.);

**Keywords:** mesh antenna, anti-pillow effect, chiral structure, NPR materials, curvature homogeneity, in-plane isotropy, Poisson’s ratio, Young’s modulus

## Abstract

The anti-pillow effect of mesh antennas has adverse effects on satellite communication. The curvature isotropy of a negative Poisson’s ratio material is expected to be applied and solved for the anti-pillow effect of mesh deployable antennas. Based on the tension characteristics of mesh antennas, our research group has proposed a novel pre-wound six-ligament chiral material, and provided the analytical solutions of Poisson’s ratio and Young’s modulus under the assumption of a small deformation. Following on from the above work, this paper takes into account the variable curvature deformation of pre-wound ligaments and the bending deformation of straight ligaments. The analytical solutions of Poisson’s ratio and Young’s modulus under large deformations are derived, and verified by finite element simulation combined for both small and large deformations. The results show that theoretical solutions considering large deformation of the ligament are more consistent with the simulation results in the large-strain range of anisotropy in the material plane. The analytical solution of Young’s modulus derived from the energy equivalent principle of elastic deformation with a curved beam and a straight beam is consistent with the simulation results under large tensile strain. It has been verified that the existence of a pre-wound ligament can slow down the deformation of the node and reduce the loss of in-plane isotropy to a certain extent, so it is easier to maintain the negative Poisson’s ratio characteristic and maintain an excellent in-plane isotropic deformation mechanism over a larger strain range under tensile load. This characteristic proves the reliability of the prospects applying the pre-wound six-ligament chiral structure in deployable mesh antennas, which lays a theoretical foundation for the subsequent prototype.

## 1. Introduction

With the rapid development of space technology, spaceborne antennas are widely used in mobile communication, navigation systems, deep-space exploration, electronic reconnaissance, and radio astronomy [1]. They are one of the most important pieces of equipment for communication satellites, reconnaissance satellites, and other systems. There are many forms of deployable antennas, among which mesh antennas are the most widely used due to their high storage ratio, low area density, and high profile accuracy. However, due to the lack of bending stiffness of metal mesh, under an external force the surfaces cannot form an ideal paraboloid shape, instead becoming surfaces with negative Gaussian curvature that are radially convex and circumferentially concave. This phenomenon, where the mesh surface deviates from the ideal paraboloid, is called the anti-pillow effect in mesh antennas [2]. The anti-pillow effect reduces the accuracy of the reflector surface and adversely affects satellite communications [3]. Previous solutions to the anti-pillow effect in mesh antennas can be divided into two categories [4]. One is based on thin-shell moment-free theory, including the application of an external load (a uniformly distributed load or a distributed load), the optimization of the boundary shape, and the adjustment of the inner membrane ratio. The other is based on thin-shell theory, including reducing the thickness of the thin shell or decreasing the in-plane elastic modulus of the thin shell. The results of these solutions are not ideal, so it is necessary to find other solutions.

Metamaterials [5] have been extensively studied in recent years because of their extraordinary mechanical properties that are not found in nature. Negative Poisson’s ratio (NPR) materials [6] are such metamaterials, and their curvature isotropy [7] has introduced new application scenarios into the study of the anti-pillow effect in mesh antennas. Negative Poisson’s ratio materials are also called auxetic materials. When a traditional material is compressed (stretched) in the longitudinal direction, it will be stretched (compressed) in the transverse direction, while the opposite is true of NPR materials. When they are compressed (stretched) in the longitudinal direction, they will also be compressed (stretched) in the transverse direction. This abnormal behavior has produced many unique mechanical properties, such as shear resistance, indentation resistance, curvature isotropy, and energy absorption.

In 1987, Lakes [8] developed the first artificial NPR material using polyurethane foam for the first time. Since then, several NPR materials have been manufactured. Two-dimensional NPR materials include concave structures [9], chiral structures [10], rotating rigid structures [11], and perforated plate structures [12]. Some three-dimensional NPR structures have also been created, inspired by two-dimensional periodic NPR materials and the development of additive manufacturing technology [13,14,15].

For application scenarios where the direction of the force cannot be anticipated, such as lightweight sandwich panels in aviation and explosion-proof panels in military vehicles, isotropy is a significant element to be considered. Mesh antennas require a tensioning system to adhere to the ribs and form a reflecting surface, and in-plane isotropy becomes particularly critical. Poisson’s ratio in two-dimensional isotropic materials fluctuates between −1 and 1. For the three-dimensional structures, the variation range is narrower (−1, 1/2). The effective properties of periodic negative Poisson’s ratio materials can be solved by the fundamental unit problem of homogenization theory. Czarnecki [16,17,18] addressed the minimum compliance problem with an isotropic modulus tensor as the design variable, obtaining the optimal structure made of ideal heterogeneous isotropic materials with specific microstructures, demonstrating the ability to model auxiliary behavior within the subdomain of assuming negative values for the optimal Poisson’s ratio. Chiral structures have the ability to provide the same mechanical characteristics in different directions in the plane, and can provide an in-plane orthotropic response through configuration adjustment, such as anti-tetra-chiral structures [19]. Therefore, we pay special attention to chiral structures. A chiral structure [20] is composed of a node as the center, with a particular number of ligaments evenly distributed around the node, and these are also tangential to the node. Such structures can be divided into different types according to the number of ligaments (three ligaments, four ligaments, or six ligaments), and the way that the ligaments surround the node (adjacent cells arranged along the direction in which the ligament is distributed is called a chiral arrangement, and adjacent cells being arranged along the opposite direction to the ligament is called an anti-chiral arrangement).

Prall [21] first carried out numerical analysis and experimental research on the in-plane characteristics of chiral materials with six ligaments. The analytical solutions of Poisson’s ratio and Young’s modulus were provided under the premise of small linear elastic deformation. Since then, Alderson [22]’s numerical and experimental research on tetra-chiral, anti-tetra-chiral, tri-chiral, and anti-tri-chiral structures demonstrated that the Young’s modulus increases with the increase in the number of ligaments. Compared to anti-chiral structures, chiral structures had a larger Young’s modulus. The Poisson’s ratio of hexa-chiral, tetra-chiral, and anti-tetra-chiral structures was close to −1. Tri-chiral structures always showed positive Poisson’s ratio, and anti-tri-chiral structures only showed negative Poisson’s ratio in their shorter ligaments. Mousanezhad [23] applied the Castigliano theorem to derive the analytical expressions of the in-plane mechanical properties of tetra-chiral, anti-tetra-chiral, tri-chiral, and anti-tri-chiral materials based on the energy method. Due to the inconsistency of their deformation mechanisms, the deformation of tetra-chiral and anti-tetra-chiral structures is mainly tensile, while the deformation of tri-chiral and anti-tri-chiral structures is mainly bending.

Clarke [24] simulated and verified the in-plane isotropic Poisson’s ratio and Young’s modulus of a hexagonal honeycomb, quadrilateral honeycomb, triangular honeycomb, and reentrant and double V-shaped structural materials under compressive load. It was found that the in-plane isotropy of hexagonal honeycombs was significantly better than that of other structures, and six-ligament chiral material is considered to be in-plane isotropic. Zhu [25] found that the in-plane isotropy of six-ligament chiral materials was only maintained well under compressive load, but that quickly lost negative Poisson’s ratio under tensile load. Therefore, a design with wavy ligaments was proposed to improve the auxetic and in-plane isotropic behavior of standard six-ligament chiral materials under large-deformation, and to exhibit higher energy absorption efficiency. In order to further improve the mechanical behavior of six-ligament chiral materials under tensile load and to reduce the deformation of the central node, Zhu [26] proposed a layered six-ligament chiral structure. Wang [27] investigated the effect of node configuration on the mechanical properties of six-ligament chiral materials, and processed thickened-node and layered-node samples, respectively. It was shown that node reinforcement can prevent the deformation of the node in the process of ligament deformation, thus promoting the rotation mechanism and improving the negative Poisson’s ratio characteristics of six-ligament chiral materials. The effect of filling with fractal nodes is more significant than that of simply thickening nodes, although his experiment was based on compressive load, and did not discuss the deformation mechanism under tensile load.

In order to meet the characteristics required by a mesh antenna tension system and to further improve the characteristics of traditional six-ligament chiral materials that rapidly lose their negative Poisson’s ratio under tensile load, Zeng [28] proposed a novel pre-wound six-ligament chiral material. Based on the original six-ligament chiral material, a segment of circular ligament is tightly wound along the node, and the circular ligament is connected with the straight ligament along a direction tangent to the node. This structure has been proven to be able to expand the strain range that maintains its auxetic characteristics under tensile load. He [29] observed the ligament deformation mechanism of a pre-wound six-ligament chiral material during a tensile test using a DIC system. On this basis, the theoretical solutions of the Poisson’s ratio and Young’s modulus of the material were derived with the assumption of a small deformation, and these were verified by simulation experiments. However, she did not discuss the deformation mechanism of the pre-wound six-ligament chiral material under a large deformation, nor did she analyze the in-plane isotropy of the material under tensile load.

Based on the above, the in-plane deformation mechanism of a pre-wound six-ligament chiral material under a large deformation is analyzed, and the analytical solutions of its Poisson’s ratio and Young’s modulus are given based on a theoretical model under a large deformation. The in-plane isotropy of the material under tensile load is also simulated and analyzed.

## 2. Theoretical Derivation of Poisson’s Ratio and Young’s Modulus Under Large Deformation

**Figure 1:**
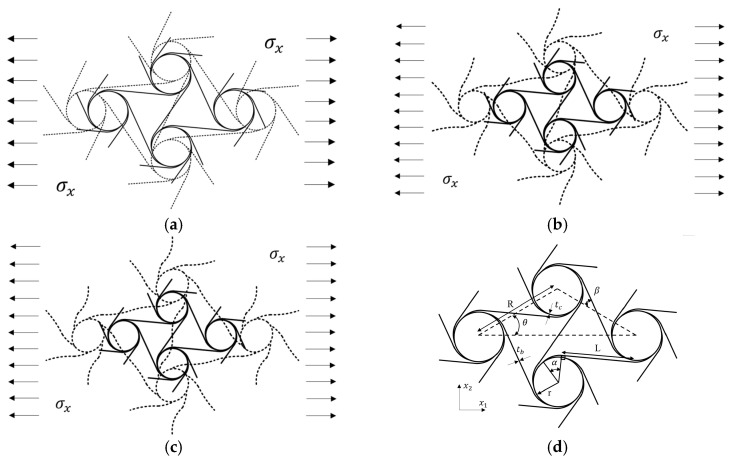
Deformation diagram of pre-wound six-ligament chiral material (**a**). Deformation diagram of the first stage (**b**). Deformation diagram of the second stage (**c**). Deformation diagram of the third stage (**d**). Parameter description of pre-wound six-ligament chiral material.

The structural parameters of the pre-wound six-ligament chiral material are presented in Figure 1d. Under uniaxial tension, it is found that the deformation of the pre-wound six-ligament chiral material can be divided into three stages: The first stage is the small deformation stage; the node rotates, the pre-wound ligament rotates and deforms inconspicuously, and the straight ligament does not experience bending deformation. It is considered that only the rigid displacement generated by rotation participates in the deformation, as shown in Figure 1a. The second stage is the large deformation stage. Under a continuous tensile load, the node’s rotation is limited to a certain extent, the gap between the pre-wound ligament and the node continues to increase, and the straight ligament is bent and deformed into an “s”-shaped curve to resist the external load, as shown in Figure 1b. Stage III: with the increase in the tensile load, the pre-wound ligament is completely pulled apart, the node cannot further rotate and deforms into an oval shape, and the uniformity of the ligament’s state of deformation is damaged. The ligament’s deformation along the direction of tension is mainly tensile, and the ligament’s deformation along the normal direction of tension is mainly bending, which leads to the material plane no longer strictly conforming to isotropy, as shown in Figure 1c.

It can be seen that the material’s deformation mode under tensile load is more complex than that under compressive load. Under compressive load, the bending of a ligament of the traditional six-ligament chiral material drives the node to rotate and partially retract into itself, making the structure more compact and the deformation mechanism more stable. The bending of the ligament complements the rotation mechanism, and the material more easily maintains the characteristics of in-plane isotropy. Under tensile load, the ligament along the main tensile direction forces the node to rotate, driving the bending deformation of the remaining ligament. With the increase in tensile stress, the deformation of the ligament along the main tensile direction is limited and drags the node until it becomes elliptical, which aggravates the loss of rotational symmetry. The large deformation of the pre-wound ligament supplements the rotation mechanism under tensile stress, slows down the node’s deformation, and makes it easier to maintain the isotropy and negative Poisson’s ratio characteristics under large strain.

### 2.1. Large Deformation Mechanism of Pre-Wound Six-Ligament Chiral Material

**Figure 2:**
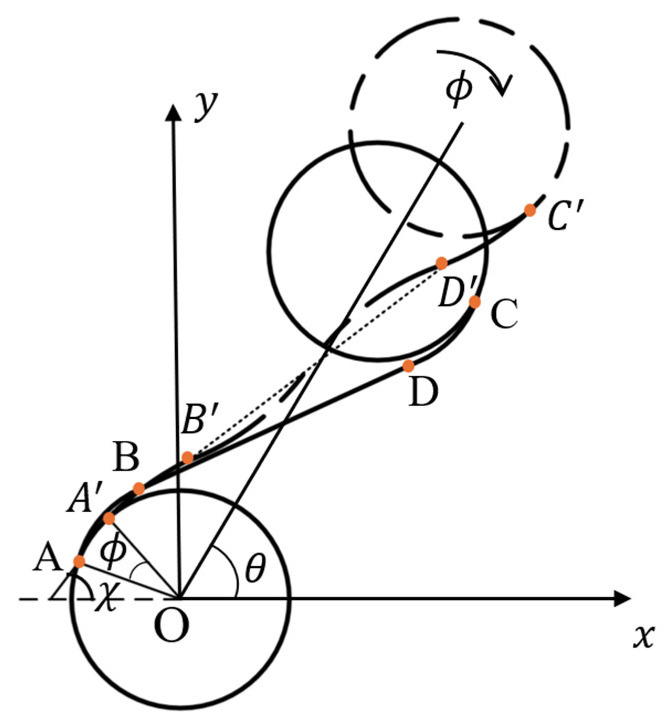
Simplified diagram of pre-wound six-ligament chiral material under large deformation.

The model of a pre-wound six-ligament chiral material under large deformation is shown in Figure 2. The solid node is in the state before deformation, and the dotted node is in the state after large deformation. The arcs AB and CD tightly wrap the node before deformation, and the curvature of the arcs $A^{\prime }B^{\prime }$ and $C^{\prime }D^{\prime }$ changes after deformation. The gap between the pre-wound ligament and the node is partly caused by the rotation of the node, and the other part is caused by the increase in the curvature of the pre-deformation arc, which shows that the pre-wound ligament is pulled apart, and the straight ligament BD is bent into an S-shape, as shown by $B^{\prime }D^{\prime }$ in Figure 2.

**Figure 3:**
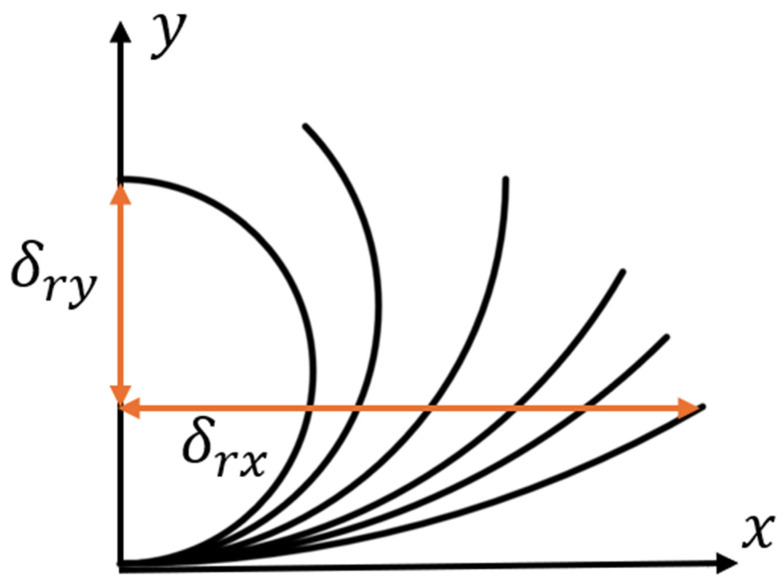
Variable arc curvature deformation.

The displacement of a single cell in the x  and y axes $e_{x},e_{y}$ can be expressed as

(1) $$\begin{array}{l} e_{x}=r\phi_{r}\cos\theta +\delta_{rx}+L\phi_{L}\cos\theta \\ e_{y}=r\phi_{r}\sin\theta +\delta_{ry}+L\phi_{L}\sin\theta \end{array}$$

r is the radius of the node, $\phi_{r}$ is the rotation angle of the node, θ is the angle between the line adjacent to the node’s center and the *x* axis. θ is always $60^{\circ }$ in the six-ligament chiral materials, *L* is the length of the straight ligament, and $\phi_{L}$ is the angular deflection of the straight ligament; $\delta_{rx},\delta_{ry}$ are the projection of the deflection of the curved beam on the x and y axes after the deformation. The deformation of the curved beam here no longer meets the assumption of a small deflection and small deformation, and the curvature has changed significantly. Figure 3 shows the displacement projection on the x and y axes after a $180^{\circ }$ arc deformation with variable curvature.

The strain $\varepsilon_{x},\varepsilon_{y}$ can be expressed as

(2) $$\begin{array}{l} \varepsilon_{x}=\frac{e_{x}}{R\cos\theta }=\frac{r\phi_{r}\cos\theta +\delta_{rx}+L\phi_{L}\cos\theta }{R\cos\theta }\\ \varepsilon_{y}=\frac{e_{y}}{R\sin\theta }=\frac{r\phi_{r}\sin\theta +\delta_{ry}+L\phi_{L}\sin\theta }{R\sin\theta } \end{array}$$

Poisson’s ratio ν can be expressed as

(3) $$\nu =-\frac{\varepsilon_{y}}{\varepsilon_{x}}=-\frac{\left(r\phi_{r}\sin\theta +\delta_{ry}+L\phi_{L}\sin\theta \right)\cos\theta }{\left(r\phi_{r}\cos\theta +\delta_{rx}+L\phi_{L}\cos\theta \right)\sin\theta }$$

### 2.2. Mechanical Model of Curved Beam with Variable Curvature

In order to describe the behavior of a pre-wound ligament under large deformation, modeling it as a curved beam made of two-dimensional elastic materials is considered, assuming that the cross-section of the curved beam does not change shape and is always orthogonal to the neutral axis in the deformation state.

**Figure 4:**
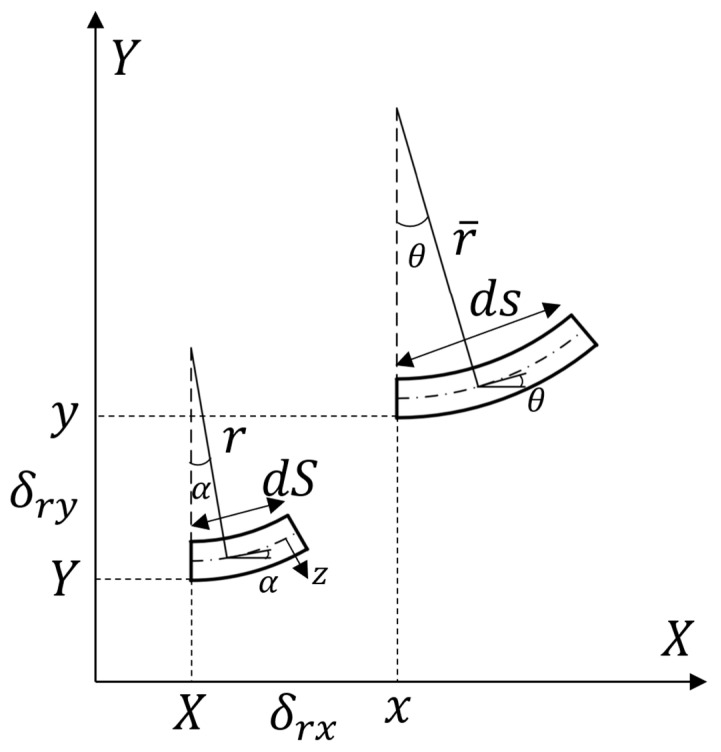
Diagram modeling two-dimensional curved beam.

For the two-dimensional curved beam structure, as shown in Figure 4, the length of the element before deformation is recorded as dS, the endpoint coordinate is (X,Y), the curvature is r, the unit length after deformation is ds, the endpoint coordinate is (x,y), and the curvature changes to $\bar{r}$ under deformation. The angle between the tangent line at any point of the curved beam and the *x* axis can prove that it is equal to the angle of the arc’s center. The inclination angle of the tangent line before deformation is α, and after deformation it is θ, with $\delta_{rx},\delta_{ry}$ representing the displacement of the arc along the x,y axis before and after deformation, respectively.

Therefore, the displacements before and after deformation have the following relationship:

(4) $$\left\{\begin{array}{l} x=X+\delta_{rx}\\ y=Y+\delta_{ry} \end{array}\right.$$

The angular deflection of the curved beam segment during deformation φ can be recorded as

(5) φ=θ−α

The strain at the neutral axis can be defined as ε=(ds−dS)/dS, and can be Simplified as ds=(1+ε)dS.

For a two-dimensional tensile curved beam, it is assumed that in-plane tension does not change the length of the neutral axis, so ε=0.

For microsegment elements of any length dS, there is a corresponding radius of curvature, and the curve before deformation meets the following requirements:

(6) dS=rdα

The curve after deformation meets

(7) $$ds=\bar{r}d\theta$$

Here, the value of the curvature’s radius is constant, representing the arc curve or other curves according to the curve characteristics, such as helix, parabola, etc.

The arc segment at the distance from the neutral axis *z* can be expressed as

(8) $$dS_{z}=(r+z)d\alpha ,ds_{z}=(\bar{r}+z)d\theta$$

The strain at *z* can be expressed as $\varepsilon_{z}=(ds_{z}-dS_{z})/dS_{z}$, and simplified as

(9) $$\varepsilon_{z}=\varepsilon +z\frac{d\varphi }{dS}$$

When z≪r, the second term in Formula (9) can be ignored; that is, when the size of the cross-section of the curved beam is far less than the radius of curvature, the strain in the direction of its thickness can be ignored, so that the axial force and bending moment are, respectively, expressed as

(10) $$N=\int_{A}E\varepsilon_{z}dA=EA\varepsilon$$

(11) $$M=\int_{A}E\varepsilon_{z}zdA=EI\frac{d\varphi }{dS}$$

**Figure 5:**
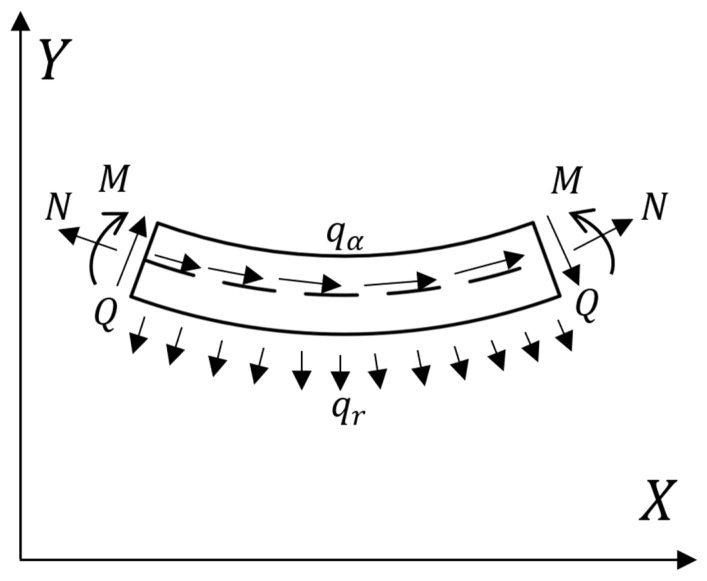
Stress microelement of curved beam.

A is the cross-sectional area, E is the Young’s modulus of the material, and I is the moment of inertia relative to the neutral axis. Assuming that I and A are constants, as shown in Figure 5, this forms a microelement of the curved beam, where N is the axial force, M is the moment, Q is the shear force, $q_{\alpha }$ is uniformly distributed tangentially to the force, and $q_{R}$ is uniformly distributed radially to the force.

The force balance equation can be written as

(12) $$\frac{dN}{dS}+\frac{Q}{r}=-q_{\alpha },-\frac{N}{r}+\frac{dQ}{dS}=-q_{R},\frac{dM}{dS}=Q$$

This is the same as the force balance equation for a small deformation. Rewrite the balance equation with the deformed configuration.

(13) $$\frac{dN}{ds}+\frac{Q}{\bar{r}}=-\frac{q_{\alpha }}{1+\varepsilon },-\frac{N}{\bar{r}}+\frac{dQ}{ds}=-\frac{q_{R}}{1+\varepsilon },\frac{dM}{ds}=\frac{Q}{1+\varepsilon }$$

So far, there are three force equations, Equation (13), and two constitutive equations, Equations (10) and (11). In order to analyze the deformation, the displacement equation is considered as

(14) dx=(1+ε)cosθdS,dy=(1+ε)sinθdS

Integrate for (14), considering (4), and the displacement $\delta_{rx},\delta_{ry}$ can be obtained.

There is no distributed load on the material under stress, which corresponds to Formula (12) $q_{\alpha }=0,q_{R}=0$.

(15) $$\frac{dN}{ds}+\frac{Q}{\bar{r}}=0,-\frac{N}{\bar{r}}+\frac{dQ}{ds}=0,\frac{dM}{ds}=Q$$

Introduce Equation (7) into the first two terms in (15) and solve it by integration as follows:

(16) $$N=A_{1}\cos\theta +A_{2}\sin\theta ,Q=A_{1}\sin\theta -A_{2}\cos\theta$$

**Figure 6:**
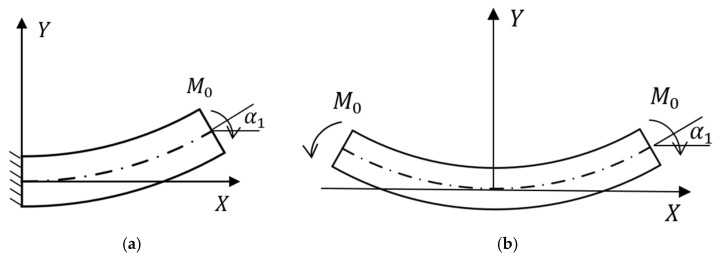
Simplified stress diagram of pre-wound ligament. (**a**) curved beam fixed at one end; (**b**) symmetrical free curved beam.

$A_{1},A_{2}$ is constant, and is determined by the boundary conditions. For the stress of the ligament under tensile conditions, it can be simplified as shown in Figure 6, where $M_{0}$ is opposite to the direction in Figure 5. Therefore, by introducing the Q expression in Equation (16) into the third equation in Equation (15), we can get

(17) $$M=-A_{1}y+A_{2}x-A_{3}$$

Here we can get x,y by integrating $x=\int_{0}^{\theta }\bar{r}\cos\theta d\theta ,y=\int_{0}^{\theta }\bar{r}\sin\theta d\theta$.

The inclination angle θ after deformation can be expressed as

(18) $$\frac{d\theta }{dS}=\frac{1}{r}-\frac{A_{1}y}{EI}+\frac{A_{2}x}{EI}-\frac{A_{3}}{EI}$$

Integrate Equation (18) to obtain

(19) $$\theta =\alpha -\frac{A_{3}}{EI}S-A_{1}\int_{0}^{\alpha }\frac{yR}{EI}d\alpha +A_{2}\int_{0}^{\alpha }\frac{xR}{EI}d\alpha$$

If the free curved beam is symmetrical relative to the y-axis shown in Figure 6 and the curved beam that is fixed along one side are equivalent, Figure 6a can be simplified as Figure 6b. From the fixed end α=0 to the free end $\alpha =\alpha_{1}$, a concentrated dimensionless moment $M_{0}$ is applied at the end of the curved beam, and the axial force, shear force, and moment at the free end $\alpha =\alpha_{1}$ can be expressed as

(20) $$N(\alpha_{1})=0,Q(\alpha_{2})=0,M(\alpha_{3})=M_{0}$$

Introduce the boundary condition (20) into Equation (16) to get the value of the constant term $A_{1}=A_{2}=0,A_{3}=M_{0}$; introduce this into (19) to get the inclination angle after deformation

(21) $$\theta =\alpha -kS,k=\frac{M_{0}}{EI}$$

(22) $$x=\int_{0}^{\alpha }r\cos(\alpha -kS)d\alpha ,y=\int_{0}^{\alpha }r\sin(\alpha -kS)d\alpha$$

Integrate (22) to obtain

(23) $$x=\frac{r}{1-kr}\sin\left(\alpha -kr\alpha \right),y=-\frac{r}{1-kr}\left[1-\cos\left(\alpha -kr\alpha \right)\right]$$

The relationship between the curvature radius after deformation and that before deformation can be obtained by

(24) $$\bar{r}=\frac{r}{1-kr}$$

where r is constant, the curvature after deformation is still constant under the action of moment—that is, the deformation of the curved beam is still an arc after being stressed, but the radius of curvature changes—and the radius of curvature becomes $\frac{1}{1-kr}$ times the initial value.

(25) $$X=\int_{0}^{\alpha }r\cos\alpha d\alpha ,Y=\int_{0}^{\alpha }r\sin\alpha d\alpha$$

Introduce (22) and (25) into Equation (4) and sort out the deformation displacement expression as follows:

(26) $$\begin{array}{l} \delta_{rx}=x-X=\int_{0}^{\alpha }2r\sin(\alpha -\frac{1}{2}kS)\sin\frac{kS}{2}d\alpha \\ \delta_{ry}=y-Y=\int_{0}^{\alpha }2r\sin(\frac{1}{2}kS)\cos(\alpha -\frac{1}{2}kS)d\alpha \end{array}$$

**Figure 7:**
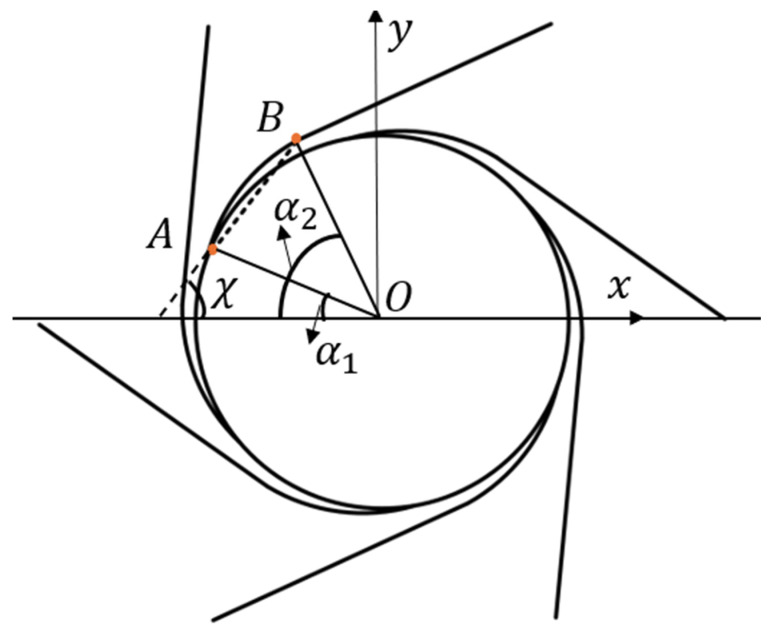
Schematic diagram of distribution angle of pre-wound ligament.

Complete the integration as follows:

(27) $$\delta_{rx}={\left.r\left[\sin\alpha -\frac{\sin(\alpha -kr\alpha )}{1-kr}\right]\right|}_{0}^{\alpha },\delta_{ry}={\left.r\left[\cos\alpha -\frac{\cos\left(\alpha -kr\alpha \right)}{1-kr}\right]\right|}_{0}^{\alpha }$$

where $\delta_{rx},\delta_{ry}$ represent the displacement changes along the x and y directions after the curvature of the curved beam under a moment. The distribution of the ligament is shown in Figure 7. The angle between the arc AB and the *x* axis is χ, and the angle between the arc AB and the *y* axis is $\frac{\pi }{2}-\chi$. The pre-wound ligament can be regarded as the part of the integral segment α of the above circular parameter equation. The pre-wound ligaments at different positions only need change the upper and lower limits of the integral, and the value α is determined by the arc of the center of the pre-wound ligament.

(28) $$\delta_{rx}={\left.r\left[\sin\alpha -\frac{\sin(\alpha -kr\alpha )}{1-kr}\right]\right|}_{\alpha_{1}}^{\alpha_{2}},\delta_{ry}={\left.r\left[\cos\alpha -\frac{\cos\left(\alpha -kr\alpha \right)}{1-kr}\right]\right|}_{\alpha_{1}}^{\alpha_{2}}$$

For the ligament distribution shown in Figure 7, the geometric relationship can be obtained by

(29) $$\alpha_{1}=\frac{\pi }{2}-\chi -\frac{\alpha }{2},\alpha_{2}=\frac{\pi }{2}-\chi +\frac{\alpha }{2}$$

It is noted that based on classical beam theory, the angular deflection of the straight ligament and the curved ligament can be expressed as

(30) $$\phi_{L}=\frac{M_{0}L}{6E_{s}I},\phi_{r}=\frac{M_{0}r\alpha }{E_{s}I},\theta =60^{\circ }$$

Introducing (30) and (28) into (3), the expression of Poisson’s ratio for analysis of curved beams with variable curvatures can be obtained as

(31) $$\nu =-\frac{6M_{0}r^{2}\alpha (1-kr)+M_{0}L^{2}(1-kr)+EIr\left[-8\sqrt{3}\cos\chi \sin\frac{\alpha }{2}(1-kr)+8\sqrt{3}\sin\left[\left(1-kr)(\frac{\pi }{2}-\chi \right)\right]\sin\left[(1-kr)\frac{\alpha }{2}\right]\right]}{6M_{0}r^{2}\alpha (1-kr)+M_{0}L^{2}(1-kr)+EIr\left[24\sin\chi \sin\frac{\alpha }{2}(1-kr)-24\cos\left[\left(1-kr)(\frac{\pi }{2}-\chi \right)\right]\sin\left[(1-kr)\frac{\alpha }{2}\right]\right]}$$

where $k=\frac{M_{0}}{E_{s}I}$.

All the unknowns in Formula (31) have been explained. Considering the complex form and difficulty of eliminating the moment, the numerical solution is obtained by introducing different geometric parameters.

### 2.3. Young’s Modulus

It is observed that the elastic deformation of the curved beam and the rigid displacement of the straight beam are involved in the material’s deformation under a small deformation (within 5% strain), but this situation is no longer consistent with the deformation mechanism under a large deformation. The elastic deformation of both the curved beam and the straight beam is also involved, resulting in an increase in the Young’s modulus of the material, which shows that the material is difficult to “pull apart” compared with its behavior under a small deformation. The Young’s modulus is deduced by the method of energy.

Considering only the bending of the ligament, ignoring the shear and tensile effects, the energy of the circular ligament can be expressed as

(32) $$U_{r}=\int \frac{M^{2}}{2E_{s}I}rd\alpha =\frac{M^{2}r\alpha }{2E_{s}I}$$

The arc’s angular deflection during tension is

(33) $$\phi_{r}=\frac{\partial U}{\partial M}=\frac{Mr\alpha }{E_{s}I}$$

Bring Equation (33) into Equation (32), and note that the section is a square, and the moment of inertia is $I=\frac{1}{12}t^{3}d$, where t is the width of the ligament in plane, d is the thickness of the material along the outside plane, and both sides of the arc are subjected to moment at the same time, so the strain energy of the curved beam is

(34) $$U_{r}=\frac{2E_{s}I\phi^{2}}{2r\alpha }=\frac{E_{s}t^{3}d\phi^{2}}{12r\alpha }$$

For a straight beam, the strain energy can be expressed as

(35) $$U_{rib}=\int Md\phi$$

The angular deflection of the beam can be obtained from the basic beam theory as follows:

(36) $$\phi_{L}=\frac{ML}{6E_{s}I}$$

Bring Equation (36) and the moment of inertia into Equation (35). Note that both sides of the straight beam are subjected to a moment, and the strain energy of the straight beam can be obtained by

(37) $$U_{L}=\frac{E_{s}t^{3}d}{2L}\phi^{2}$$

**Figure 8:**
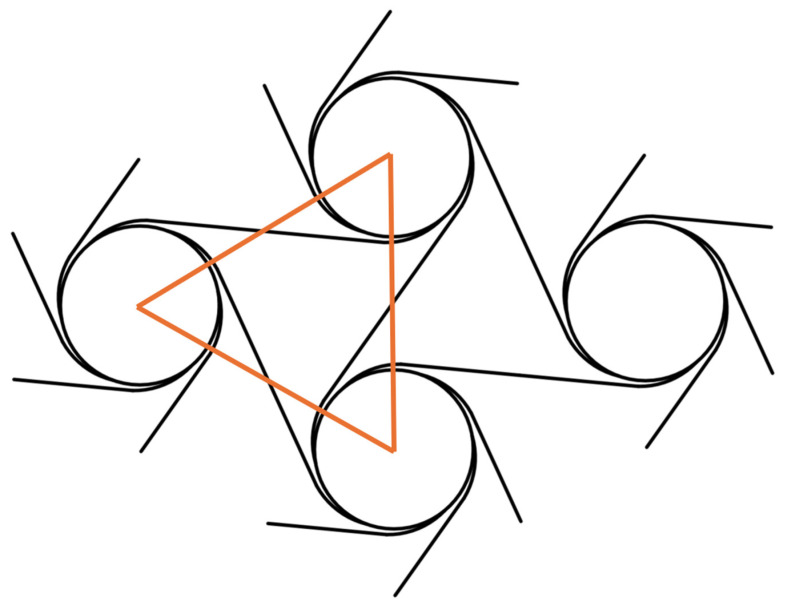
Schematic diagram of feature unit.

Take the triangle as the characteristic element, as shown in Figure 8, and each characteristic element contains 3 curved beams and 1.5 straight beams, so the total strain of the characteristic element can be expressed as

(38) $$U_{r}=\frac{E_{s}t^{3}d}{4r\alpha }\phi^{2}+\frac{3E_{s}t^{3}d}{4L}\phi^{2}$$

where $\phi \approx \frac{\varepsilon R}{r}$.

In homogenization mechanics, $U=V\int \sigma d\varepsilon =\frac{1}{2}EV\varepsilon^{2}$, where $V=\frac{\sqrt{3}}{4}R^{2}d$.

According to the conservation of energy, the Young’s modulus of the material under a large deformation can be expressed as

(39) $$\frac{E}{E_{s}}=\frac{4\sqrt{3}t^{3}}{3r^{3}\alpha }+\frac{4\sqrt{3}t^{3}}{Lr^{2}}=\frac{4\sqrt{3}}{3}\frac{1}{\alpha }\frac{t^{3}}{r^{3}}+4\sqrt{3}\frac{t^{3}}{L^{3}}\frac{L^{2}}{r^{2}}$$

The equivalent Young’s modulus of the material under a large deformation is divided into two items. The first item is related to the pre-wound ligament, which is inversely proportional to the pre-wound angle and is proportional to ${\left(t/r\right)}^{3}$, and the second item is related to the straight ligament, which is proportional to ${\left(t/L\right)}^{3}$, confirming that bending is the dominant behavior of the straight ligament.

It can be seen that the increase in the pre-wound ligament undoubtedly reduces the Young’s modulus of the material (the pre-wound angle α in the expression is on the denominator), making the material easier to pull apart. The energy distribution of the traditional six-ligament chiral material is mainly the bending elasticity of ligaments ($\propto {\left(t/L\right)}^{3}$) [21]. The energy of the pre-wound six-ligament structure under a large deformation is distributed into two parts: one part is distributed in the elastic deformation of the pre-wound ligament (proportional to $\propto {\left(t/r\right)}^{3}$, and inversely proportional to α), and the other part is distributed in the bending deformation of the straight ligament ($\propto {\left(t/L\right)}^{3}$). Therefore, the increase in the pre-wound ligament does change the energy distribution of the structure’s tensile deformation [30].

## 3. In-Plane Isotropy of Pre-Wound Six-Ligament Material Under Large Deformation

**Figure 9:**
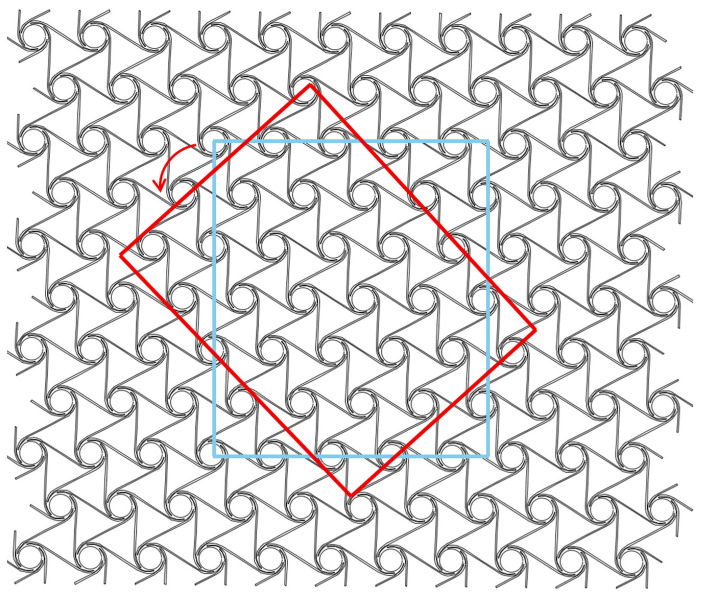
Schematic diagram of cell sample rotation.

As shown in Figure 9, the position circled by the blue frame in the material cell is the initial angle, recorded as 0 degrees (the line connecting the center points of adjacent nodes coincides with the x axis), and each 5 degrees of rotation counterclockwise is a sample model, which is imported into the simulation software for analysis, and the Poisson’s ratio data of the material in all directions are recorded.

In order to check the isotropy of a plane, four material configurations—a 15° pre-wound six-ligament material with L/R = 0.83 (L = 12), a 30° pre-wound six-ligament material with L/R = 0.83 (L = 12), a 45° pre-wound six-ligament material with L/R = 0.81 (L = 12), and a 60° pre-wound six-ligament material with L/R = 0.8 (L = 12), are rotated and trimmed, respectively. Draw Poisson’s ratio under different tensile directions and different tensile strains in the plane.

**Figure 10:**
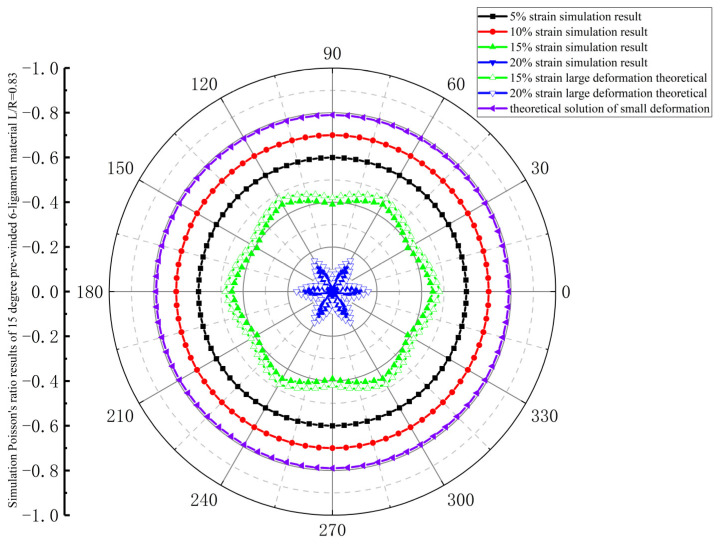
Simulation results of Poisson’s ratio for 15-degree pre-wound six-ligament material with L/R = 0.83.

As shown in Figure 10, the Poisson’s ratio of the 15-degree pre-wound six-ligament chiral material under 5% and 10% tensile strain is displayed as a circle in polar coordinates when in-plane tensile stress in all directions was applied to the material, which means the Poisson’s ratio of the material is consistent and isotropic in this plane. When the material strain increases from 5% to 10%, the absolute value of the Poisson’s ratio gradually increases. This process is due to the pre-wound ligament deforming with different curvatures, and the gap between the pre-wound ligament and node being pulled apart. This gap supplements the tensile strain margin of the traditional six-ligament chiral material. When the material strain increases to 15%, the Poisson’s ratio is no longer consistent when tensile stress is applied in different directions in the plane, and the absolute value of Poisson’s ratio decreases compared with the absolute value at strains of 5% and 10%, which shows that the absolute value of the material’s Poisson’s ratio in the direction of the connecting line between the center points of adjacent nodes is larger than that at other positions. This is because the angle between the direction in which the force is applied and the distribution direction of the pre-wound ligament is at its smallest. When the pre-wound ligament is completely pulled apart, the axial force component along the ligament is relatively large, and the contribution of the deflection of the slender beam along the axial force is very small. Therefore, the root of the ligament and the connection of the node will be deformed along the direction of the stress, and the node will become elliptical, and the in-plane isotropy will be destroyed. With the increase in strain, the 15-degree pre-wound six-ligament chiral material with L/R = 0.83 loses in-plane isotropy at a tensile strain of 15%.

**Figure 11:**
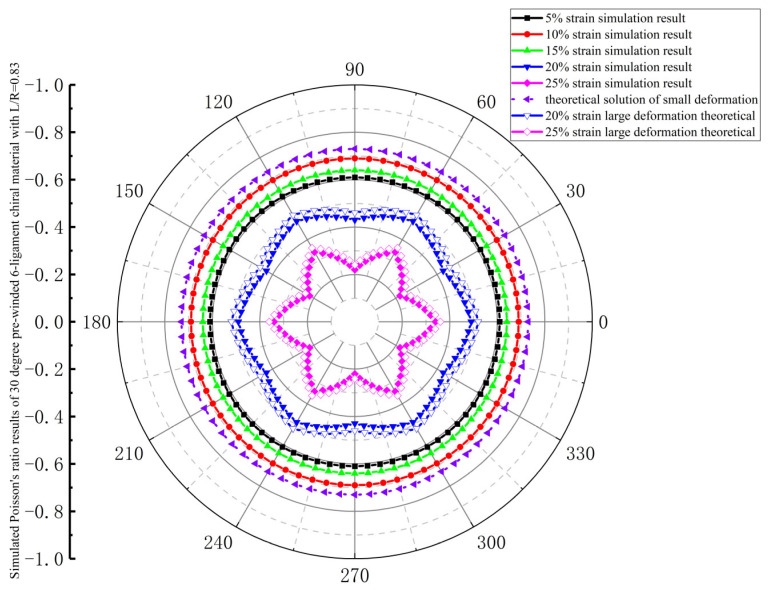
Simulated Poisson’s ratio results of 30-degree pre-wound six-ligament chiral material with L/R = 0.83.

The in-plane isotropy during the deformation process of a 30-degree pre-wound six-ligament chiral material is shown in Figure 11. The Poisson’s ratio of stress applied in all directions in the plane is consistent under strains of 5%, 10%, and 15%, which can be considered as in-plane isotropy. In the range of 5–10% strain, the absolute value of Poisson’s ratio increases with the increase in tensile strain. In the range of 10–15% strain, the absolute value of Poisson’s ratio decreases with the increase in tensile strain, but it is still larger than the absolute value of Poisson’s ratio at a 5% strain. When the strain reaches 20%, the Poisson’s ratio is no longer circular, and the in-plane isotropy is destroyed. When the strain reaches 25%, the in-plane anisotropy increases, and the absolute value of Poisson’s ratio decreases sharply. It can be considered that a 30-degree pre-wound six-ligament chiral material with L/R = 0.83 exhibits in-plane isotropy up to a tensile strain of 15%.

**Figure 12:**
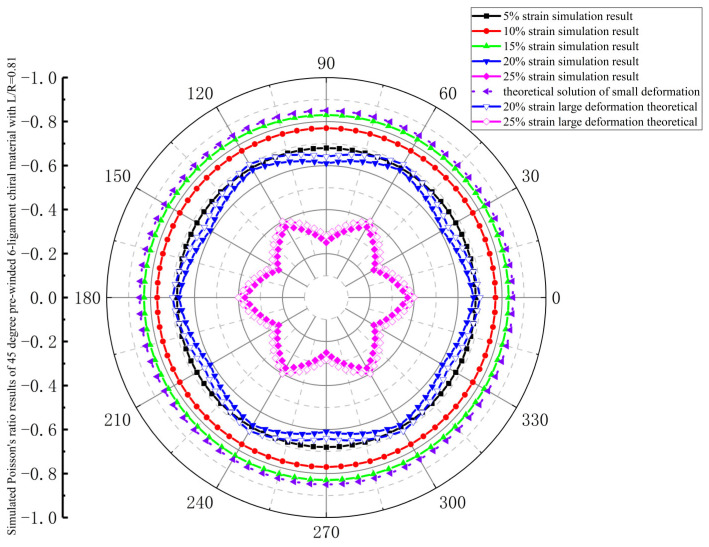
Simulated Poisson’s ratio results of 45-degree pre-wound six-ligament chiral material with L/R = 0.81.

As shown in Figure 12, the in-plane isotropy of the 45-degree pre-wound six-ligament chiral material with L/R = 0.81 is similar to that of the 30-degree pre-wound six-ligament chiral material, which exhibits in-plane isotropy at tensile strains of 5%, 10%, and 15%. However, unlike the 30-degree pre-wound chiral material, the absolute value of Poisson’s ratio increases with the increase in strain in the range of 5–15% and gradually approaches −1. At a tensile strain of 20%, in-plane anisotropy occurs, the absolute value of Poisson’s ratio decreases to its value at a tensile strain of 5%, and the in-plane anisotropy increases when the strain increases to 25%.

**Figure 13:**
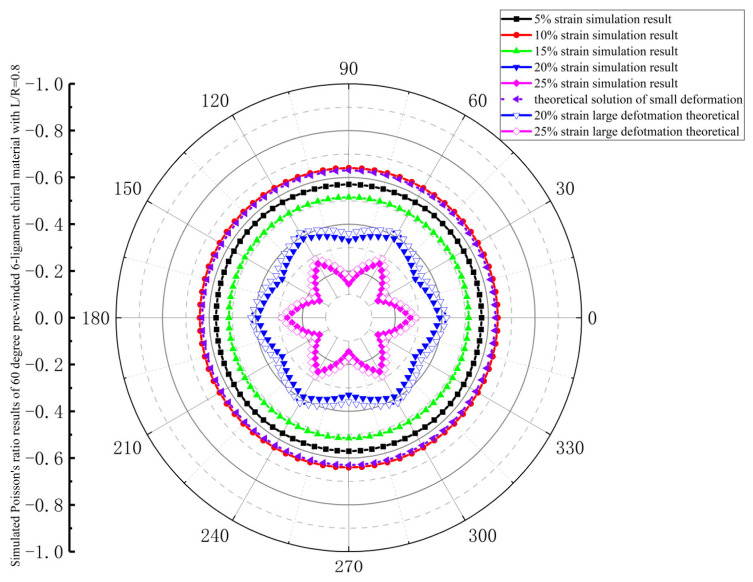
Simulated Poisson’s ratio results of 60-degree pre-wound six-ligament chiral material with L/R = 0.8.

The in-plane isotropic consistency of 60-degree pre-wound six-ligament chiral material is shown in Figure 13. The simulation value of Poisson’s ratio is smaller than that of the 30-degree and 45-degree pre-wound chiral materials, and in the range of 5–15%, with the increase in strain, the absolute value of Poisson’s ratio first increases and then decreases, and the in-plane anisotropy begins at a tensile strain of 20%, and increases at 25%.

**Figure 14:**
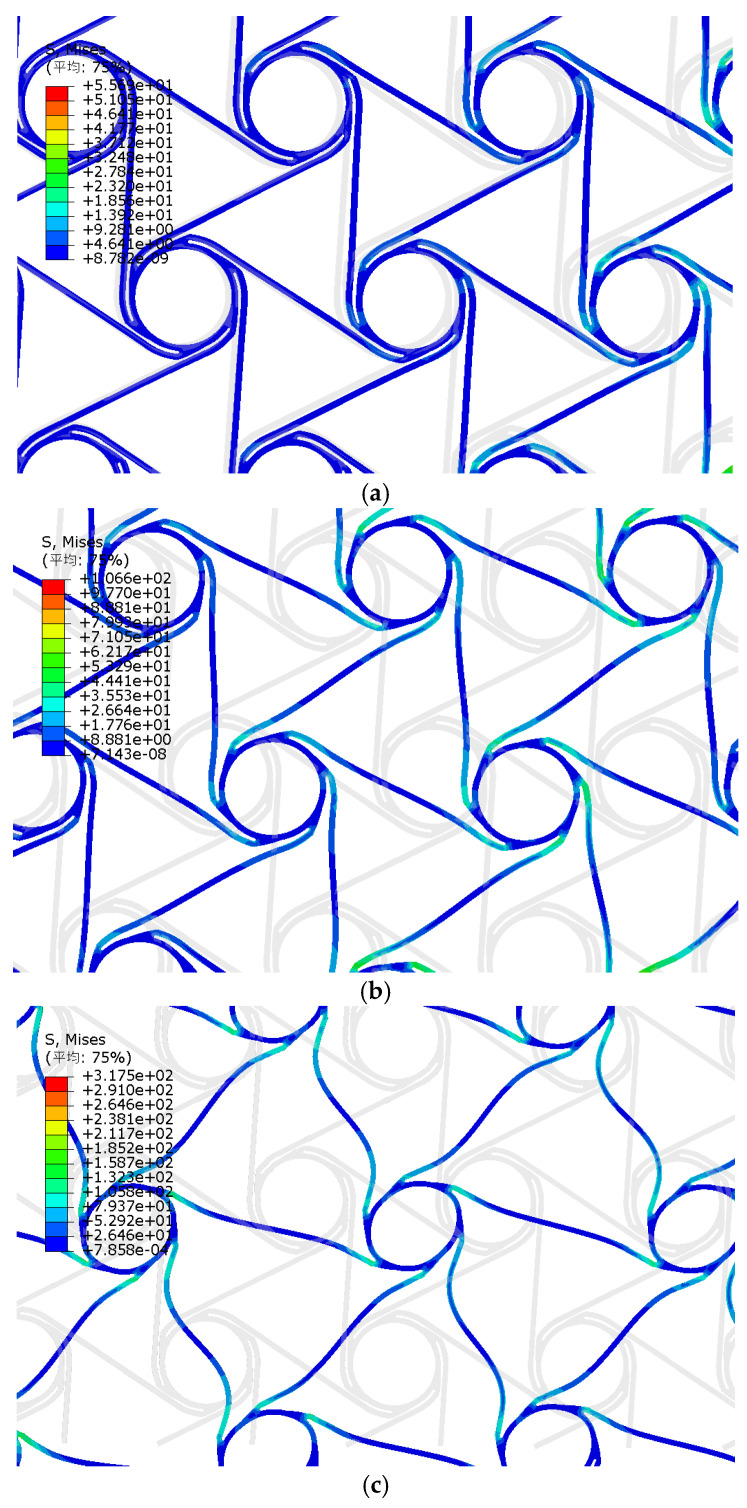
Deformation state simulation of pre-wound six-ligament chiral structure. (**a**) deformation stage one; (**b**) deformation stage two; (**c**) deformation stage three. (The Chinese characters in the upper left corner mean average).

In the simulation, the deformation process of the ligament can be observed visually, as shown in Figure 14a,b. Under tensile stress, the rotation of the nodes drives the deformation of the pre-wound ligament and the straight ligament. As the tensile strain increases, the pre-wound ligament is gradually pulled apart to compensate for the deformation mechanism of the material. At this time, only the rigid displacement of the straight ligament participates in the deformation. As the tensile strain continues to increase, the pre-wound ligament is fully unfolded, and the rotation of the node is also restricted to some extent. The straight ligament bends and deforms to resist the tensile load, as shown in Figure 14c. This is consistent with the deformation phenomena observed experimentally in the literature [29]. Therefore, it is proven that the simulation process is accurate.

The pre-wound six-ligament chiral structure exhibits in-plane 6-fold symmetry. Since the deflection contributed by the variable curvature of the pre-wound ligament is larger than that of node rotation and straight ligament bending, the projection of the deflection contributed by the variable curvature circular ligament along the in-plane X and Y axes will change with the angle between the pre-wound ligament and the X and Y axes, which leads to the Poisson’s ratio obtained by introducing the deflection of the variable curvature of the pre-wound ligament not being in-plane isotropic, and the in-plane anisotropy will increase with the increase in the material’s tensile strain. Therefore, the derivation of the variable curvature of the pre-wound ligament to the Poisson’s ratio under a large deformation is more consistent with the simulation results for in-plane anisotropy. When the tensile strain is small, the theoretical solution for a small deformation that is available in the literature [29] is more consistent with the simulation results.

**Figure 15:**
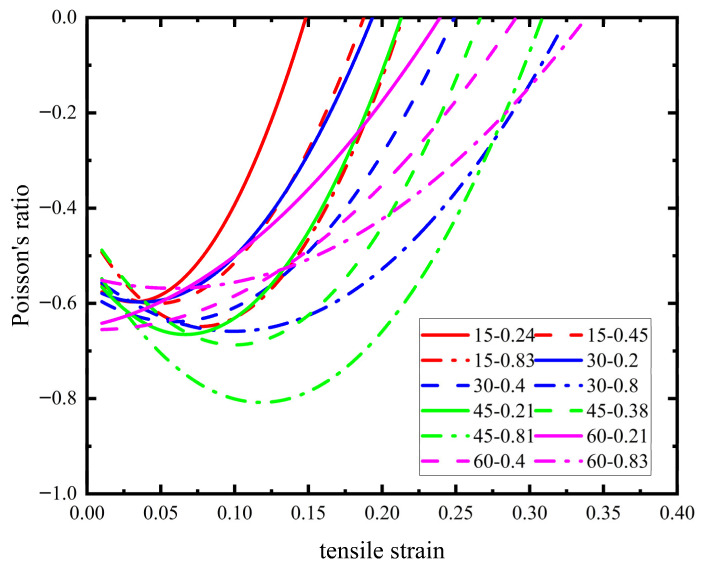
Poisson’s ratio of pre-wound six-ligament chiral materials with different parameter structures.

In order to compare the Poisson’s ratio of samples with different parameters, the Poisson’s ratio values of materials with different parameters are obtained by simulating the material samples with the polar coordinate angle of 0 degrees, as shown in Figure 15.

When the pre-wound angle is determined, the Poisson’s ratio will first decrease and then gradually increase with the increase in L/R until the negative Poisson’s ratio characteristic is lost. This is because the pre-wound ligament is gradually pulled apart, and with the increase in the straight ligament L, the gap between the pre-wound ligament and node is more fully pulled apart, until a certain value where the pre-wound ligament is fully deformed, and the straight ligament is also bent to its limit. At this point, the node’s deformation will participate as a continuous increase in strain, and the negative Poisson’s ratio of the material will gradually decrease until it completely disappears.

**Figure 16:**
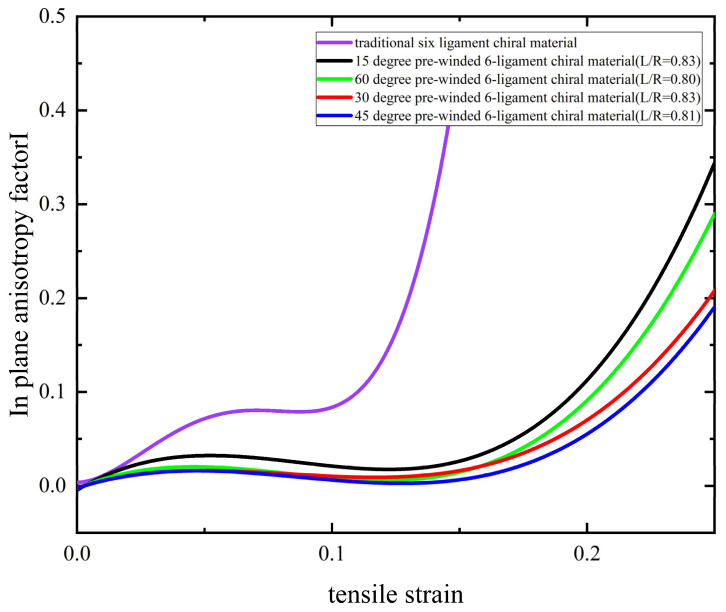
In-plane anisotropy factor of pre-wound six-ligament chiral materials with different parameters.

In order to further quantify the in-plane isotropy of such materials, the in-plane anisotropy factor ϖ is introduced as follows:

(40) $$\varpi =\frac{\left|\nu_{\max}-\nu_{\min}\right|}{\left|\nu_{\max}\right|+\left|\nu_{\min}\right|}$$

where $\nu_{\max}$ and $\nu_{\min}$ are the maximum and minimum Poisson’s ratios across 360 degrees in the plane under a given strain, and the value range of ϖ is 0–1. The closer it is to 1, the higher the in-plane anisotropy, and the closer it is to 0, the better the in-plane isotropy. The values of the in-plane anisotropy factors of pre-wound six-ligament chiral materials with different parameters and the traditional six-ligament chiral material under different strains are shown in Figure 16. It can be found that the pre-wound six-ligament chiral material has better in-plane isotropy over a larger tensile strain range than the traditional six-ligament chiral material, which proves that the improvement of the pre-wound ligament makes the material more suitable for an mesh antenna structure under tension. The increase in the pre-wound angle can improve the negative Poisson’s ratio characteristics to a certain extent, making them closer to the theoretical limit of −1. However, a large pre-wound angle may wrap around nodes too much, which limits the rotation of the nodes to a certain extent, and is not conducive to the realization of the negative Poisson’s ratio limit value.

The material’s critical strain threshold for the transition from isotropic to anisotropic mechanical behavior is related to the value of the in-plane anisotropy factor I. If the requirements of in-plane isotropy are strict, then the corresponding definition of the in-plane anisotropy factor I is smaller, and the critical strain threshold range for different parameters is more stringent; conversely, the critical strain threshold value is more relaxed. In the case of a small deformation, the deformation mechanism of the material is the node’s rotation, the rigid displacement of the straight ligament, and the stress deformation of the pre-wound ligament. In the case of a large deformation, the curvature of the pre-wound ligament changes, and the straight ligament also experiences elastic deformation. Therefore, using the control variable method, under the same conditions, by only changing the value of L/R, the critical strain threshold for the transition to in-plane anisotropy will also increase with the increase in the straight ligament L, and by only changing the pre-wound angle, the critical strain threshold for the transformation into in-plane anisotropy will increase with the increase in the pre-wound angle.

## 4. Young’s Modulus of Pre-Wound Six-Ligament Chiral Material Under Large Deformation

**Figure 17:**
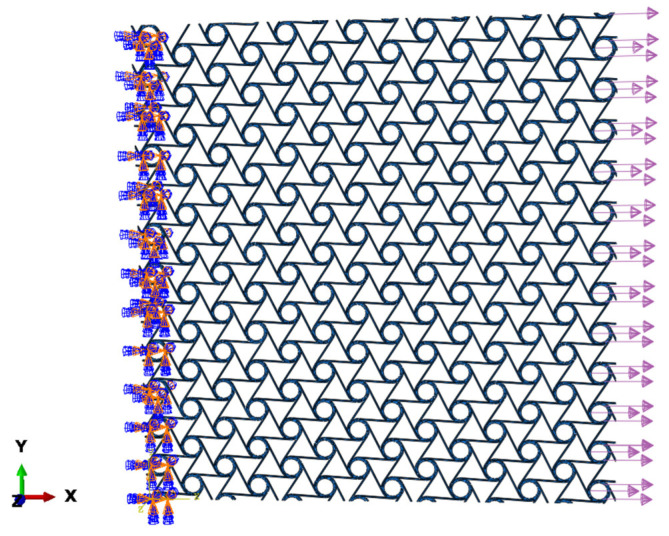
Schematic diagram of cell simulation.

As shown in Figure 17, tensile load is applied on the right side of the cell, and a fixed constraint is applied on the left side. The equivalent stress σ and strain ε of the cell can be expressed as

(41) $$\sigma =\frac{F}{S}=\frac{F}{l_{y}d}$$

(42) $$\varepsilon =\frac{l_{x}-l_{x}^{\prime }}{l_{x}^{\prime }}$$

**Figure 18:**
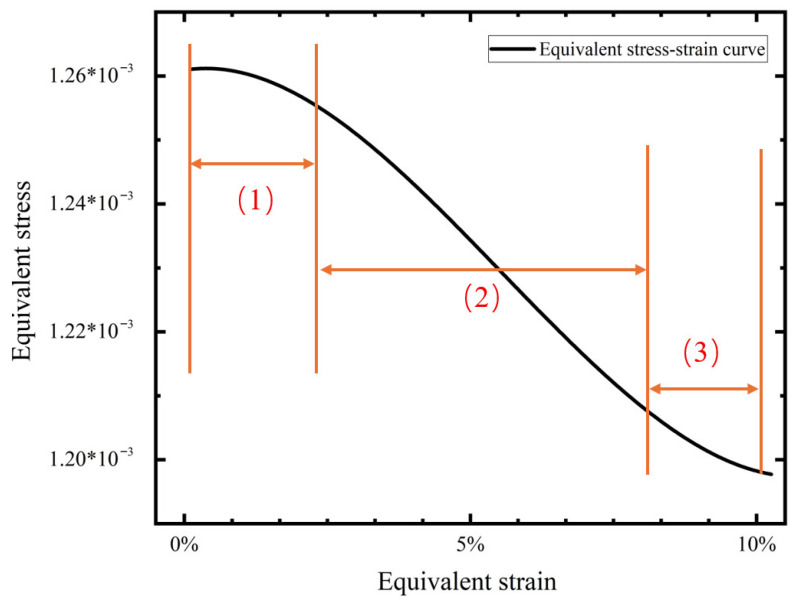
Equivalent stress–strain diagram.

F is the force exerted on the right cell, $l_{x},l_{y}$ is the cell length along the x and y axes after the material is stressed, and $l_{x}^{\prime }$, $l_{y}^{\prime }$ is the initial length of the cell in the x and y directions. Based on the above model, the equivalent stress and strain of the cell sample can be statistically obtained, as shown in Figure 18.

In order to distinguish Young’s modulus under large and small deformations more accurately, the slope of the stress–strain curve is calculated by taking the interval of the smaller strain, and the slope of stress–strain curve is equal to Young’s modulus. The stress–strain curve can be divided into three parts: The first part corresponds to the small deformation stage. Under tensile load, node rotates, the pre-wound ligament is not obviously pulled apart, the straight ligament does not appear to experience elastic deformation, and only the rigid displacement participates in the material’s deformation. The second part corresponds to the large deformation stage. With the increase in tensile load, the gap between the pre-wound ligament and the node is obviously opened. The elastic deformation of the straight ligament participates in the resistance to the tensile load, and the slope increases gradually. In the third part, the tensile load is further increased, the deformation is more intense, and the node is also involved in the deformation, so the material soon loses its negative Poisson’s ratio characteristic.

In this paper, only the value of the equivalent Young’s modulus under a negative Poisson’s ratio is recorded, and the material’s Young’s modulus without a negative Poisson’s ratio under a large deformation is no longer recorded.

**Figure 19:**
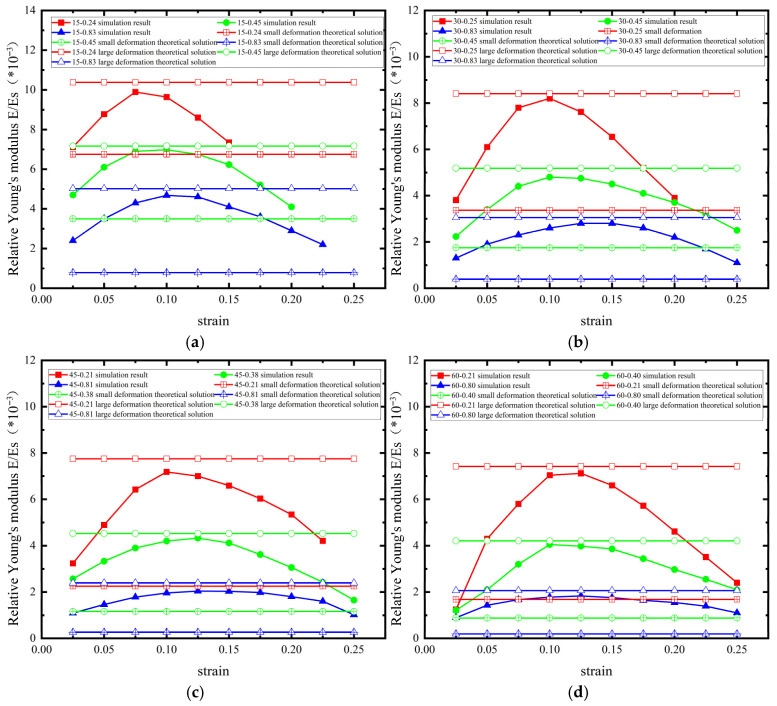
Equivalent Young’s modulus of pre-wound 6-ligament chiral materials with different parameters ((**a**) 15° pre-wound (**b**) 30° pre-wound (**c**) 45° pre-wound (**d**) 60° pre-wound).

The relative Young’s modulus of the six-ligament material with a pre-wound angle of 15 degrees is shown in Figure 19a. The rigid displacement and elastic deformation of the straight ligament supplement the deformation margin under tensile load in the microscopic mechanism of the material’s deformation. With the increase in L/R, the geometric proportion of the straight ligament’s structure inside the material increases, broadening the range of the tensile strain of the material so that it maintains its negative Poisson’s ratio characteristics at the macro scale. Under the geometric structures L/R = 0.24, 0.45, and 0.83, the value of the equivalent Young’s modulus is close to the theoretical solution for a small deformation, as available in the literature [29], when the strain is small. As the strain gradually increases, the equivalent Young’s modulus increases to the vicinity of the theoretical solution for a large deformation, then it gradually decreases unitl the negative Poisson’s ratio is lost. The curve of the equivalent Young’s modulus is similar to a parabola. When L increases, the rigid displacement under a small deformation increases, corresponding to the growth in the first stage in Figure 18. In Young’s modulus, the apex of the parabola moves to the right with the increase in L. At the same pre-wound angle, the deviation between the theoretical solutions for a large deformation and a small deformation becomes smaller with the increase in L, and the curve becomes more gentle.

The equivalent Young’s modulus of the 30-degree pre-wound six-ligament chiral material is shown in Figure 19b. The trend of the change in Young’s modulus is similar to that of the 15-degree pre-wound six-ligament chiral material. The difference is that the span of the transverse axis of the curve increases as the angle of the pre-wound ligament increases, which is consistent with the pre-wound ligament widening the range of the tensile strain of the material and maintaining its negative Poisson’s ratio characteristics at the macro scale. The 45-degree pre-wound chiral material has a similar rule to that of the 30-degree pre-wound chiral material. With the increase in L, the bending deformation of the straight ligament can bear a greater load. As the second stage deformation in Figure 18 increases, the apex of the parabolic curve in Figure 19b becomes more gentle. This can be seen in the different L/R configurations with pre-wound angles of 15 degrees, 30 degrees, 45 degrees, and 60 degrees of (as shown in Figure 19a–d).

With the increase in pre-wound angle, the theoretical value of equivalent Young’s modulus decreases whether it is large deformation or small deformation, indicating that the existence of pre-wound ligament can make the material easier to pull apart. It is verified that the existence of pre-wound ligament improves the performance of the original material under tensile load. The greater the L/R, the smaller the difference between the estimated values of Young’s modulus under a small deformation and a large deformation. The smaller the L/R, the greater the difference between the analytical solutions of Young’s modulus in the above two models. Therefore, in order to reduce the difference in Young’s modulus between large and small deformations, the material structure with large pre-wound angle and a large L/R should be selected.

## 5. Conclusions

In this paper, the deformation mechanism of a pre-wound six-ligament chiral material under a large deformation is analyzed, and analytical solution is given for Poisson’s ratio considering the deformation of a pre-wound ligament with a variable curvature. Based on the energy homogenization method, the analytical solution of the Young’s modulus of the material under a large deformation is derived, and the simulation results under different parameters are verified. The deformation mechanism of the auxetic and isotropic properties of the pre-wound six-ligament chiral material is largely affected by the rotation of the central node. The research shows that the existence of the pre-wound ligament can slow down the deformation of the node and reduce the loss of in-plane isotropy to a certain extent. Therefore, the pre-wound six-ligament chiral material can maintain a negative Poisson’s ratio and excellent in-plane isotropy across a larger strain range under tensile load. This characteristic proves the reliability of the application prospects of the pre-wound six-ligament chiral material in deployable mesh antennas, and lays a theoretical foundation for the subsequent prototype.

## Data Availability

The original contributions presented in this study are included in the article. Further inquiries can be directed to the corresponding author.
